# What Do We Still Need to Know? Pressing Issues and Promising Directions in Research on Perfectionism and Nonsuicidal Self-injury

**DOI:** 10.3389/fpsyg.2022.873410

**Published:** 2022-05-18

**Authors:** William F. Janssen, Chloe A. Hamza

**Affiliations:** Ontario Institute for Studies in Education, University of Toronto, Toronto, ON, Canada

**Keywords:** self-injury, perfectionism, risk factor, mental health, future research

## Introduction

Research investigating the compulsive, overwhelming need to be perfect (and to expect perfection from others) has burgeoned over the past three decades (e.g., Curran and Hill, 2019). This work has demonstrated that perfectionistic behavior, particularly overly harsh evaluations of performance, is often associated with elevated risk for mental health concerns (e.g., depressive symptoms, anxiety; e.g., Limburg et al., 2017) and poor health outcomes (e.g., cardiovascular illness; Molnar et al., 2012). One mental health concern that has received comparatively less attention in the literature in relation to the need to be perfect is *nonsuicidal self-injury* (NSSI). NSSI refers to deliberate, self-inflicted alteration or damage to one's body tissue without suicidal intent (International Society for the Study of Self-injury, 2018). Although existing research on the link between perfectionism and NSSI has been well summarized (see Gyori and Balazs, 2021), clearly defined priorities for future research in this area are lacking. In the present paper, we underscore why additional research is necessary and offer several specific promising research directions.

## Perfectionism and NSSI

*Perfectionism* is the setting of and striving toward exceptionally high and unrealistic standards, combined with frequent thoughts about attaining those standards and engaging in overly critical evaluation of one's performance (Frost et al., 1990). Broadly, the “umbrella term” of perfectionism has been used to encompass many different facets of the construct, including perfectionism as a stable personality disposition (i.e., trait perfectionism), as well as more state-like perfectionism (e.g., perfectionism cognitions). Trait perfectionism, which has been most extensively studied, is typically regarded as multidimensional. Two latent higher-order dimensions have been identified that encompass the variance across different scales measuring trait perfectionism: perfectionistic strivings and perfectionistic concerns (Stoeber and Otto, 2006). *Perfectionistic strivings* (PS) involves setting of and striving toward excessively high standards and goals for oneself (Dunkley et al., 2006). *Perfectionistic concerns* (PC) is the experience of holding high standards, constant punitive self-evaluation, inability to feel satisfied with objectively successful performance, and persistent concerns about the criticism and disapproval of others (Dunkley et al., 2006; Sirois and Molnar, 2017). Another comparatively less studied, more state-like feature of perfectionism includes *perfectionism cognitions*, which describe a cognitive process that involves persistent and automatic thoughts about the need to be perfect, often arising in situations in which there is clear evaluation or in which one perceives “failure” in their performance (Flett et al., 2016). Perfectionism differs from similar constructs such as self-criticism or conscientiousness in that the individual strives for an unwavering standard of perfect performance and avoidance of errors (a striving that is not an inherent feature of these other constructs), and anything other than this unattainable standard is perceived as a “failure” (Powers et al., 2004).

Research suggests that perfectionism is a transdiagnostic risk factor (Egan et al., 2011), implicated in the development and maintenance of a variety of mental health concerns (Limburg et al., 2017), including depressive symptoms, anxiety, and disordered eating (e.g., Kawamura et al., 2001; Sherry et al., 2004; Damian et al., 2017). More recently, perfectionism has been implicated in NSSI. In a recent systematic review of 15 studies (see Gyori and Balazs, 2021), it was found that perfectionism–particularly PC–was associated with increased risk for NSSI. However, studies in this review were overwhelmingly cross-sectional, and there is a need for research to clarify the processes through which perfectionism may lead to NSSI. Theory on perfectionism underscores that there may be common pathways from trait perfectionism to psychopathology, including through eliciting heightened stress (i.e., stress generation) and social isolation (i.e., social disconnection model), as a result of the individual striving for extremely high, unrealistic standards for the self (e.g., Hewitt and Flett, 2002; Hewitt et al., 2006; Smith et al., 2020). There also may be unique processes through which perfectionism may exacerbate risk for NSSI, which have not yet been examined.

**Figure 1:**
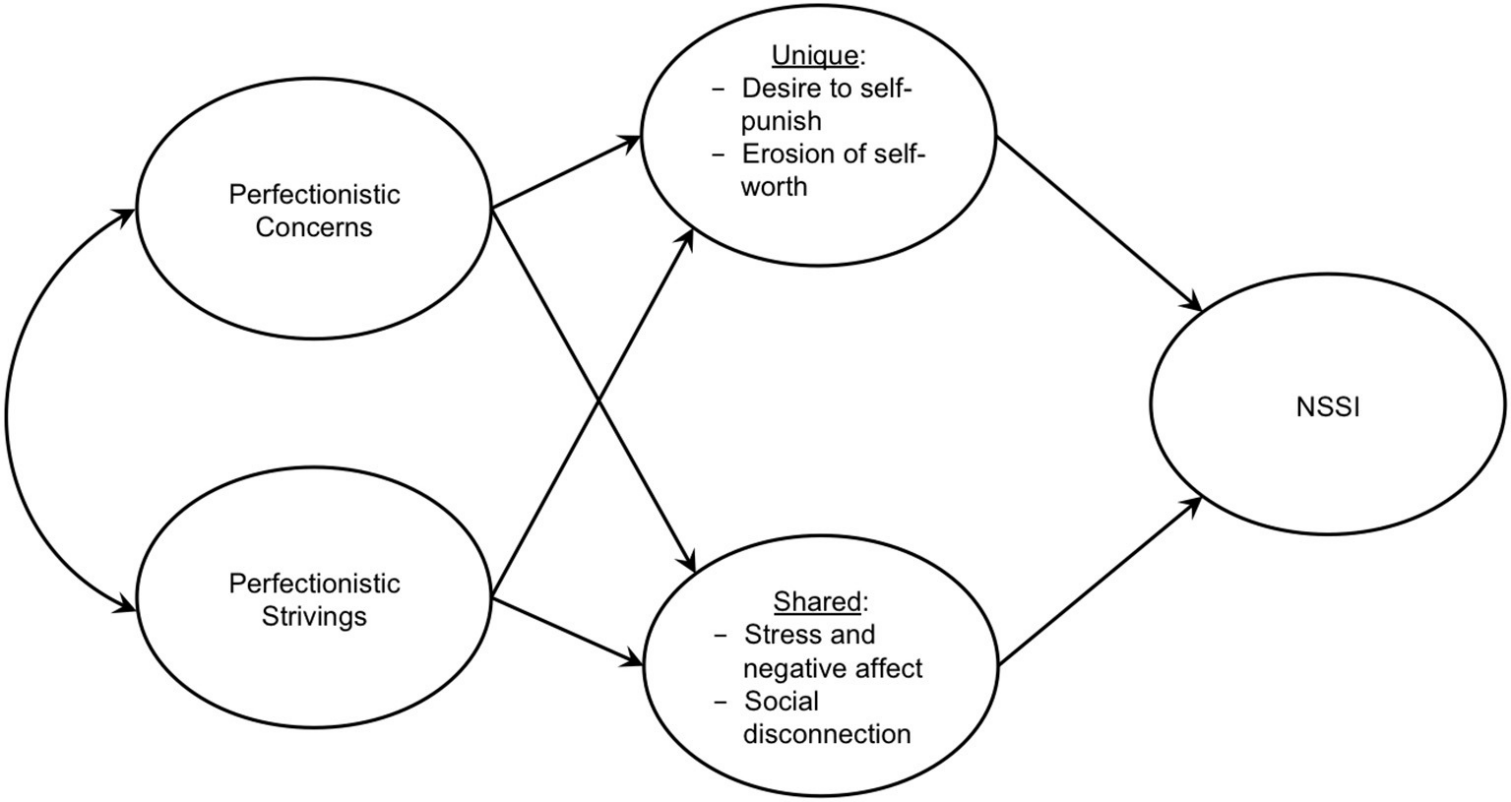
Proposed Pathways from Trait Perfectionism to NSSI. We propose that perfectionism uniquely exacerbates risk for NSSI *via* heightened desire to self-punish and erosion of self-worth, as well as through common pathways established for other mental health concerns. It is likely that the link from perfectionism to NSSI is also moderated by other risk factors for NSSI (e.g., low aversion to pain).

In a recent conceptual model of NSSI, Hooley and Franklin (2018) underscored that specific “barriers” may prevent individuals from accessing universal “benefits” for NSSI, such as emotion regulation and gratification of self-punishment–with self-punishment being a particularly unique “benefit” of NSSI. In this model, one of these key “barriers” to NSSI is self-worth, as high self-worth is thought to prevent individuals from self-directed anger and harm (Hooley and Franklin, 2018). Within the context of this theory, perfectionism may make one of the unique “benefits” of NSSI (i.e., gratifying the need for self-punishment) more desirable, while at the same time eroding the self-worth barrier (e.g., continuously perceiving to have failed to meet one's expectations/standards leading to more negative self-beliefs)–see Figure 1. Although views toward the self have long been underscored in the etiology of NSSI (Nock, 2009), researchers have to examine the processes through which perfectionism and NSSI are associated. To extend research in this area we now highlight several important directions for future research.

## Pressing Issues and Promising Research Directions

As highlighted in our proposed model in Figure 1, first and foremost there is a need for researchers to consider the multidimensional nature of trait perfectionism, as well as the extent to which dimensions of perfectionism may be differentially related to NSSI. Past research has often failed to disentangle associations between PC, PS, and NSSI. Further, it is possible that PS may lead to PC over time, thus leading indirectly to heightened risk for NSSI. In one study on perfectionism and NSSI, Claes et al. (2012) reported a positive correlation between PS and PC, which suggests that individuals who are high in PS might also engage in the extremely harsh self-evaluative patterns of thinking that are characteristic of PC. This is why studying the interconnectedness between these dimensions of perfectionism over time, and their impacts on NSSI, is essential.

Given the cross-sectional nature of the research on trait perfectionism and NSSI (e.g., Hoff and Muehlenkamp, 2009; Flett et al., 2012; Lucas et al., 2019), there is also a pressing need to understand the processes through which perfectionism may exacerbate risk for NSSI. In our model, we propose that perfectionism may uniquely exacerbate risk for NSSI by simultaneously: (a) increasing the desire to self-punish, and (b) eroding self-worth. Research has yet to examine how perfectionism is related to different functions for engaging in NSSI functions for engaging in NSSI, although in one study authors found that PC was correlated with self-punishment motivations (Claes et al., 2012). Individuals who are highly self-critical (e.g., individuals who score highly on PC) may engage in NSSI as a form of self-punishment in an effort to express self-directed anger resulting from feeling dissatisfied with their performance or their perceived failure. These desires to self-punish may be particularly salient when the self-worth barrier has eroded due to perfectionistic tendencies or thoughts (i.e., repeated failures to meet perfectionistic standards). We also anticipate that perfectionism will exacerbate risk for NSSI through heightened stress, negative affect, and social disconnection–akin to other mental health concerns (Ashby et al., 2011; Smith et al., 2020). Indeed, NSSI has been shown to serve emotion regulatory and interpersonal coping “benefits” in addition to self-punishment (Taylor et al., 2018).

Perfectionism cognitions may also be relevant to NSSI. A comparatively more state-like facet of perfectionism, perfectionism cognitions may be associated with salient moment-to-moment thoughts about needing to attain perfection (and self-derogation for perceiving to fail to meet an unattainable standard of performance) that could motivate NSSI engagement to cope with these thoughts as they arise. Although there is a robust link between trait perfectionism and the experience of perfectionism cognitions (e.g., Frost et al., 1997), extant literature suggests that perfectionism cognitions explain unique variance in mental health outcomes over and above that explained by trait perfectionism (e.g., Flett et al., 2007). Experiential sampling approaches (e.g., ecological momentary assessment [EMA]) that capture real-time changes in perfectionism cognitions and NSSI could address this pressing gap in the literature. EMA has already been used to study NSSI, with several studies (e.g., Nock et al., 2009; Armey et al., 2011) supporting the notion that elevated negative affect precedes most instances of NSSI engagement.

In addition to testing the proposed pathways, researchers should examine potential moderators, such as exposure to stressful experiences (e.g., traumatic events or chronic stressors; Smith et al., 2014), which may also be important in understanding the relation between perfectionism and NSSI. For example, the association between perfectionism and NSSI may be heightened in situations in which failures are exacerbated as a result of stressful life events (e.g., failed relationship, poor academic or work performance). Further, it is likely that the link between perfectionism and NSSI is stronger among individuals with other known risk factors for NSSI (e.g., heightened pain thresholds; Kirtley et al., 2016). Gender may also be another important moderator of the link between perfectionism and NSSI (Flett et al., 2012), though further research is needed. Understanding when individuals with high levels of perfectionism are most at risk for NSSI engagement (e.g., in the context of failure) is necessary to inform targeted mental health interventions.

The use of repeated measures assessments would also help to help to clarify whether the effect of perfectionism on NSSI is unidirectional or more reciprocal in nature. It is possible that NSSI, as well as other mental health challenges (e.g., depressive symptoms), lead to a greater experience of trait perfectionism over time. For example, Asseraf and Vaillancourt (2015) found that increases in depressive symptoms lead to increases in trait PC over time among youth across grades 6–8. This finding suggests that, at least in childhood, the experience of depressive symptoms may increase one's perception that others expect excessively high standards of performance, and that there is a need to satisfy this perception. With respect to NSSI, it could be the case that engaging in perfectionistic behaviors is similarly a coping strategy to compensate for engagement in NSSI or to alleviate the feelings of internalized shame and poor self-concept that may contribute to NSSI (e.g., Mahtani et al., 2019).

Future research could also continue to consider the developmental underpinnings of perfectionism. A prevailing developmental theory (Flett et al., 2002) posits that parental criticism represents a vulnerability factor in the etiology of psychopathology by contributing to the development of trait perfectionism. For example, Claes et al. (2012) found that perfectionism represented an indirect pathway between parental criticism and NSSI among females with an eating disorder (also see Yates et al.'s, 2008 study of youth). Relatedly, some researchers (e.g., Maloney et al., 2014) have reported empirical findings that parenting factors, such as parental expectations and parental criticism, are key developmental factors in the etiology of trait perfectionism. Understanding the effects of these early environmental influences on trait perfectionism, and potentially in turn NSSI, will help to illuminate the early developmental underpinnings of trait perfectionism and NSSI.

It is also noteworthy that studies on perfectionism and NSSI to date primarily rely on self-report measures (Lucas et al., 2019), which can lead to bias in the responses (e.g., responding to items in a socially desirable way; Rosenman et al., 2011). Furthermore, measures often ask for recall of behaviors over long periods (e.g., lifetime NSSI), which can be subject to recall errors (Hoff and Muehlenkamp, 2009). Lab-based measures of perfectionism, such as a text replication task (Yiend et al., 2011) or a bead-sorting task (Bouchard et al., 1999)—both of which measure aspects of perfectionism (e.g., attention to detail, checking behavior) and have been used to study behavioral perfectionism (Yiend et al., 2011)—may be one way to address some of the limitations of retrospective self-report assessments.

Finally, there is a pressing need for consideration and inclusion of more diverse samples in the literature, given that the research to date has been primarily conducted with Western, Caucasian, and female participants. There is a need to explore the relevance and cultural appropriateness of perfectionism in more culturally diverse samples (Raymundo, 2021), as well as consider the moderating effects of gender on perfectionism and NSSI. Additionally, the utility of perfectionism in the prediction of NSSI may be especially salient among clinical populations, who are receiving treatment for NSSI and thus engage in NSSI more frequently, relative to community-based samples. Moreover, some studies have found PS to be elevated within clinical samples (e.g., Egan et al., 2011), which might make this particular dimension of trait perfectionism more relevant among populations being treated for NSSI.

## Discussion

Overall, the extant literature and theory provide encouraging evidence for a potential relation between NSSI and trait perfectionism (Gyori and Balazs, 2021), but there are several important issues in the field that need to be addressed. Specifically, further research is needed to clarify the nature of the associations between perfectionism and NSSI. Conducting longitudinal research that considers the temporal associations between the multiple dimensions of trait perfectionism (i.e., PC, PS) and NSSI represents an important next step in the field. Additionally, future research should explore the potential mechanisms underlying the link between perfectionism and NSSI (e.g., *via* self-punishment desires and erosion of self-worth), as well as the moderating factors that may influence the strength of the relationship (e.g., failure, stress, gender). Future work on perfectionism and NSSI also will be strengthened through the use of more diverse methods of assessment (e.g., lab-based/objective) among more diverse samples of participants. Clearly, much remains to be known about the association between perfectionism and NSSI.

## Author Contributions

WJ and CH conceptualized the paper. WJ wrote the first draft of the manuscript. CH edited and provided feedback on the draft. All authors contributed to manuscript revision, read, and approved the submitted version.

## Funding

CH wants to acknowledge receipt of funding support from the Social Sciences and Humanities Research Council (SSHRC) to conduct this research (Grant Number: 435-2018-0961). The funder was not involved in conceptualizing or writing of the work.

## Conflict of Interest

The authors declare that the research was conducted in the absence of any commercial or financial relationships that could be construed as a potential conflict of interest.

## Publisher's Note

All claims expressed in this article are solely those of the authors and do not necessarily represent those of their affiliated organizations, or those of the publisher, the editors and the reviewers. Any product that may be evaluated in this article, or claim that may be made by its manufacturer, is not guaranteed or endorsed by the publisher.
